# Genome-Wide Association Study (GWAS) of White Mold Resistance in Snap Bean

**DOI:** 10.3390/genes13122297

**Published:** 2022-12-06

**Authors:** Haidar A. Arkwazee, Lyle T. Wallace, John P. Hart, Phillip D. Griffiths, James R. Myers

**Affiliations:** 1 Horticulture Department, College of Agricultural Engineering Sciences, University of Sulaimani, Sulaimani 46001, Iraq; 2 USDA-ARS, Plant Germplasm Introduction and Testing Research Unit, 201 Clark Hall, Washington State University, Pullman, WA 99164, USA; 3 USDA-ARS, Tropical Agriculture Research Station (TARS), 2200 P. A. Campos Ave., Suite 201, Mayagüez, PR 00680-5470, USA; 4 School of Integrated Plant Sciences (Horticulture Section), Cornell University Agritech, 635 W. North St., Geneva, NY 14456, USA; 5 Department of Horticulture, Oregon State University, 4017 Ag & Life Sciences Bldg., Corvallis, OR 97331, USA

**Keywords:** common bean, *Phaseolus vulgaris*, *Sclerotinia scleotiorum*, GWAS, SNP, GBS, FarmCPU, Middle American genome, pentatricopeptide repeat, leucine-rich repeat

## Abstract

White mold can result in snap bean yield losses of 90 to 100% when field conditions favor the pathogen. A genome-wide association study (GWAS) was conducted to detect loci significantly associated with white mold resistance in a panel of snap bean (*Phaseolus vulgaris* L.) cultivars. Two populations of snap bean were used in this study. The first population was the BeanCAP (Coordinated Agriculture Project) Snap Bean Diversity Panel (SBDP) (*n* = 136), and the second population was the Snap Bean Association Panel (SnAP) (*n* = 378). SBDP was evaluated for white mold reaction in the field in 2012 and 2013, and SnAP was screened in a greenhouse only using the seedling straw test in 2016. Two reference genomes representing the Andean and Middle American centers of domestication were utilized to align the genotyping-by-sequencing (GBS) data. A GWAS was performed using FarmCPU with one principal component after comparing five models. Thirty-four single-nucleotide polymorphisms (SNPs) significantly associated with white mold resistance were detected. Eleven significant SNPs were identified by the seedling straw test, and 23 significant SNPs were identified by field data. Fifteen SNPs were identified within a 100 kb window containing pentatricopeptide repeat (PPR)-encoding genes, and eleven were close to leucine-rich repeat (LRR)-encoding genes, suggesting that these two classes are of outsized importance for snap bean resistance to white mold.

## 1. Introduction

White mold, caused by *Sclerotinia sclerotiorum* (Lib.) de Bary, is considered one of the most economically important diseases affecting common bean (*Phaseolus vulgaris* L.) in temperate growing environments [1]. White mold is a soilborne, necrotrophic, and non-host-specific fungus that infects >400 plant species. In common bean, both the grain legume form (dry bean) and the vegetable form (snap or green bean) are strongly affected by this disease. In addition to reducing yield and quality in the field, snap bean harvest lots with >3% moldy pods may be rejected at the processing plant. Several cultural practices can be used to manage white mold in bean fields, such as decreased plant density, orientation of rows to prevailing winds, surface rather than overhead irrigation, the application of fungicides such as Fluazinam, and the use of biological controls such as *Coniothyrium minitans* or *Trichoderma* spp. [2,3]. However, the most effective strategy to reduce the disease severity and decrease the use of fungicides is breeding beans for host-plant resistance to white mold [4].

The genetic control of resistance to white mold can be partitioned into physiological resistance and disease avoidance [5]. Avoidance traits may allow for escape from the disease through early maturity before conditions are favorable for pathogen development or may create an unfavorable environment for the pathogen through altered growth habit and plant architecture. Disease avoidance includes traits such as canopy porosity, lodging resistance, reduced flower number, glabrous stems and leaves, and increased cuticle thickness [5]. Plant architecture is controlled by both qualitative and quantitative factors and may be influenced by environmental conditions [1,5]. Physiological resistance to white mold in common bean appears to involve traditional pathogen defense pathways, in addition to oxalate tolerance [6,7]. Whereas some researchers have reported Mendelian inheritance, most have found quantitative resistance for physiological resistance [4,8,9].

Many white mold resistance and avoidance quantitative trait loci (QTL) have been reported for common bean, most with a small to moderate effect and residing in all linkage groups except Pv10 [1]. Twenty-seven QTL for disease resistance and 36 for avoidance have previously been identified in multiple biparental populations in independent studies using various testing methods [5]. Previous research on 14 recombinant, inbred, biparental populations involving 12 dry bean and two snap bean parents identified 37 QTL associated with resistance. A meta-analysis of these populations revealed 17 distinct QTL [9]. Recent genome wide association studies (GWAS) have refined the QTL to smaller intervals and identified new QTL [10].

GWAS is a powerful genomics tool developed to detect the genes responsible for traits of interest in humans, animals, and plants [11]. Several GWAS statistical models have been developed, including the general linear model (GLM), which considers fixed effects only, and the mixed linear model (MLM), which includes both fixed and random effects [12]. Fixed and random circulating probability unification (FarmCPU) model appears to be particularly effective in controlling the false-positive error rate, which can be a problem with GLM, while reducing false negatives caused by excessive stringency of the MLM [13]. In addition to FarmCPU, other last-generation GWAS models that improve type I and type II statistical errors include Bayesian-information and Linkage-disequilibrium Iteratively Nested Keyway (BLINK) and Settlement of MLM Under Progressively Exclusive Relationship (SUPER), which are generally considered to be comparable with FarmCPU [14].

GWAS models must typically account for population structure to avoid a bias for false positives derived from population structure. Population structure in common bean is primarily determined by two regions of domestication: the Middle American center and the Andean center [15]. Reproductive barriers exist between these two centers of domestication, which results in the expression of dwarf lethal alleles, seed unviability, and physiological abnormalities that are manifested when members belonging to one center of domestication are hybridized with members of the other center [16]. Dry beans have largely maintained separation between the centers of domestication, but snap bean has undergone considerable hybridization, resulting in a high degree of admixture between these two distinct genetic populations [17,18]. Significant differences exist between the Middle American and Andean centers of domestication in terms of disease resistances and the genes that underlie domestication [5,19].

The objective of this research is to better understand the genetic basis of white mold resistance in snap beans by identifying QTL derived from (1) phenotypes from both a greenhouse setting and a field setting (2) using both a Middle American and Andean reference genome to assess novel QTL that may be present in only one reference genome. Snap beans have undergone selective pressures and extensive inter-gene pool introgression distinct from dry beans; the identification of these QTL will facilitate marker-assisted breeding in snap beans for white mold resistance, providing new sources of resistance for dry beans.

## 2. Materials and Methods

### 2.1. Populations

Two populations of snap bean were used in this study. The Bean CAP (Coordinated Agriculture Project) Snap Bean Diversity Panel (SBDP) [20] includes 150 cultivars and breeding lines, of which 136 determinate (bush) accessions were evaluated in this study (Table S1). The second population termed the Snap Bean Association Panel (SnAP) consists of 378 cultivars and breeding lines, of which 376 were evaluated in this study (Table S2) [21]. The SBDP was developed prior to and is included within the SnAP. The SBDP was evaluated for white mold resistance in the field in 2012 and 2013. The SnAP was screened for white mold resistance in the OSU greenhouses using the seedling straw test in 2016 [22,23]. The partially resistant check, G122, was included in all experiments for comparison but was not genotyped.

### 2.2. Phenotyping: Field

Field evaluations were carried out at the OSU Vegetable Research Farm located near Corvallis, Oregon, on the lower bench of the Willamette River floodplain on a Chehalis silty clay loam soil at latitude 44.572722, longitude −123.241351 at 77 MASL. Most fields on the farm have a history of moderate to high risk of disease when snap beans are grown, and soils carry a significant load of white mold sclerotia when environmental conditions favor the disease.

Seeds were planted at a depth of approximately 5 cm in single-row plots with a length of 3.0 m, 77 cm between rows, and a density of 50 seeds per plot. A single row of bush blue lake cultivar OSU 5630, a white-mold-susceptible check, was planted as borders and end plots (1.5 m) to minimize edge effects. The trials were conducted in fields with a history of disease to ensure natural inoculation during the growing season. Plots were irrigated on a weekly basis with approximately 2.5 cm applied through solid-set overhead sprinklers. At flowering, irrigation was shifted to daily application in late afternoon or early evening for ~30 min to increase leaf wetness duration and create a more favorable microenvironment for disease development.

Incidence and severity were measured in three replicates arranged in a randomized complete block design. Incidence was measured as percentage of diseased plants within each plot. Severity was measured by observing diseased plants and rating symptoms using a 1–9 scale as follows: 1 = no disease symptoms on main stem, branches, or pods; 2 = lesions starting on main stem, branch, and/or pods; lesion < 2 cm located in internodal regions; 3 = lesion reaching a node; 4 = lesion passing through a node and extending approximately 2 to 3 cm; 5 = lesion spanning two nodes; 6 = lesion passing through two nodes; 7 = lesion passing through three nodes; 8 = lesion reaching four nodes; and 9 = lesion passing through four nodes or more (or whole-plant collapse). This scoring method was modified in year two to score incidence based on any sign of disease and to score the most diseased plant in the plot for severity. A disease severity index (DSI) was calculated as the geometric mean of incidence and severity for a given year (Table S1). An average of years for severity and incidence was also calculated (Table S1). Owing to weather and field conditions, disease pressure was more severe in the second year.

### 2.3. Phenotyping: Greenhouse (Seedling Straw Test)

The SnAP was evaluated for white mold reaction in the greenhouse using a modified straw test [24] designated the seedling straw test [22,23]. The trial was repeated to obtain three biological replicates with four technical replicates within each biological replicate. Owing to the relatively large population size, one replicate at a time was evaluated, with the complete evaluation taking approximately three months. Four seeds per line were planted in 10 cm pots in soilless media (Sungro Horticulture, Agawam, MA, USA) supplemented with 3.5 g Osmocote 14-14-14 slow-release fertilizer (Scotts Miracle-Gro Co., Marysville, OH, USA). For inoculation, actively growing mycelia from fungal sclerotia (JRS isolate #152 collected from a dry bean field in Mitchell, Nebraska) were produced on potato dextrose agar. A plug of agar that included the outer edge of the mycelia was taken using a soda straw segment to inoculate 7-to-10-day-old seedlings. With the modified method, the stems were cut 1–2 cm above the primary leaves, and a straw with two plugs of agar with fungal mycelia was placed on the decapitated stem. Seedlings were scored four days after inoculation using a 1–9 scale: 1 = no lesion, 3 = lesion reaching first node, 5 = lesion reaching halfway between the first and cotyledon nodes, 7 = lesion reaching cotyledonary node, 9 = seedling completely collapsed and dead.

### 2.4. Genotyping

The original SnAP population was genotyped by sequencing (GBS) at the Cornell University Institute of Biotechnology Genomic Diversity Facility [21]. A total of 25,472 SNPs were generated after 0.01 minor allele frequency (MAF) filtering in alignment with G19833, v2.1, a reference genome of primarily Andean derivation. A total of 34,442 SNPs were generated after 0.01 MAF filtering in alignment with 5-593, v1.1, a reference genome of primarily Middle American derivation. SNPs with 40% or more missing values were eliminated. The methods of alignment and imputation are otherwise identical to those described by Saballos et al. (2022) [25]. Because the SBDP is a subpopulation of the SnAP, a subset of the GBS data extracted from the original resource (SnAP) was used for all association studies using the SBDP.

### 2.5. Data Analysis

All data sets were tested for normality with a Shapiro–Wilk test in the R statistical software environment, version 4.2.1. The average severity for 2012 and 2013 and the average of incidence for 2012 and 2013 were normally distributed (Table S3). The index (geometric mean) of severity and incidence for 2012 was normally distributed, but the index of severity and incidence for 2013 was left-skewed, and no transformation could bring about normality. The straw test data were right-skewed, but a log transformation resulted in normally distributed data (Table S3). Although not utilized in the GWAS, the data for severity in 2012, severity in 2013, incidence in 2012, and incidence in 2013 were examined and found to be not normally distributed (Table S3).

Analysis of variance (ANOVA) implemented in R ver. 3.3.2 was conducted for phenotypic data for both field and greenhouse tests. The linear additive model for the field test consisted of lines (genotype) and years as main effects, as well as the interaction of lines with years and replication nested within years. The model for the seedling straw test consisted of lines and replications as main effects.

To examine relationships among mean incidence, mean severity, the yearly index, and the greenhouse seedling straw test, Pearson’s correlation coefficients were calculated using PROC CORR in SAS (ver. 9.4, SAS Inst., Cary, NC, USA). Least square means were used for all traits as an input into geometric means and two-year averages.

### 2.6. Genome-Wide Association Study

Five models were tested utilizing the GAPIT software package, version 3, in the R statistical software environment, version 4.2.1: a mixed linear model (MLM), multiple loci mixed linear model (MLMM), Bayesian-information and Linkage-disequilibrium Iteratively Nested Keyway (BLINK), and Fixed and random model Circulating Probability Unification (FarmCPU). One, two, and three principal components (PC) were calculated in TASSEL, version 5.2.84, and tested with each of these models. Based on a visual evaluation of Q-Q plots for the best fit around the null distribution, FarmCPU with one PC axis was determined to be the best model, and all GWAS analyses were performed with this model. FarmCPU is an R package developed by Liu et al. (2016) [13] that exploits a multilocus mixed model (MLMM) and completes the analysis in two iterative steps. A fixed-effect model (FEM) is first applied, followed by a random-effect model (REM); the steps are repeated iteratively until no significant SNPs are detected. To identify SNPs significantly associated with traits, a Bonferroni (BF) threshold was calculated by dividing the probability level (α) by the number of effective markers to estimate a cutoff at α = 0.05. Effective marker numbers were used because SNPs are often correlated with each other and do not represent independent tests. The simpleM method was executed in the R software environment to calculate effective marker numbers [26]. The negative log value of α = 0.05 divided by the effective marker number yielded Bonferroni cutoffs of 4.862 for the SnAP panel and 4.705 for the SBDP panel, both with the G19833 reference genome, as well as 5.483 for the SnAP panel and 5.108 for the SBDP panel with the 5-593 reference genome. These Bonferroni cutoffs were used in all GWAS conducted in this study. The resulting associated SNPs identified by GWAS were further analyzed for their R^2^ values through a one-way ANOVA conducted using the SAS GLM procedure (SAS v9.4) with trait as the dependent variable.

## 3. Results

### 3.1. Seedling Straw Test: Analysis of Variance

An ANOVA of the seedling straw test applied to the SnAP in the greenhouse for disease severity showed that differences among lines were highly significant (*p* < 0.01). Highly significant differences were also observed among replicates (*p* < 0.01) (Table 1). The regression coefficient (R^2^) and coefficient of variation (CV) were 0.57 and 24.99, respectively.

**Figure 1:**
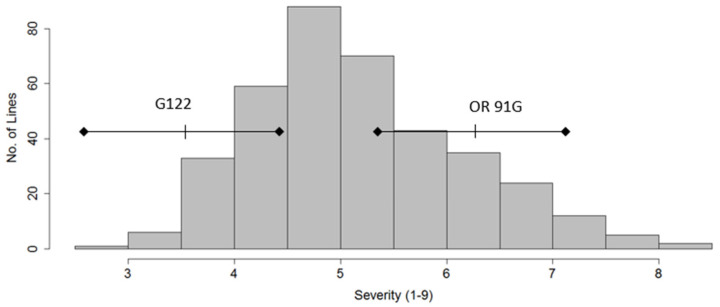
Distribution of the Snap Bean Association Panel (SnAP) lines, G122 (resistant check) and OR 91G (susceptible check), evaluated for disease severity (1–9 scale, where 1 = resistant) of white mold (*Sclerotinia sclerotiorum* (Lib.) de Bary) in the OSU greenhouse using the seedling straw test. The least square means for G122 (partially resistant) and OR 91G (susceptible) of 3.33 and 6.41, respectively, are shown with 95% confidence intervals.

Least square means (LSM) for disease severity in the straw test showed a wide range of responses to fungal inoculation from the extremely susceptible to extremely resistant (Figure 1). The LSM for disease severity for the checks G122 (resistant) and OR91G (susceptible) were 3.33 and 6.67, respectively. Few lines fell on the extremes of the distribution, with the majority of lines between the two checks. Lines with low scores were not significantly different from the resistant check, but a number of lines showed significantly higher scores compared to the susceptible check (Figure 1; Table S2).

### 3.2. Straw Test: Genome-Wide Association Study

**Figure 2:**
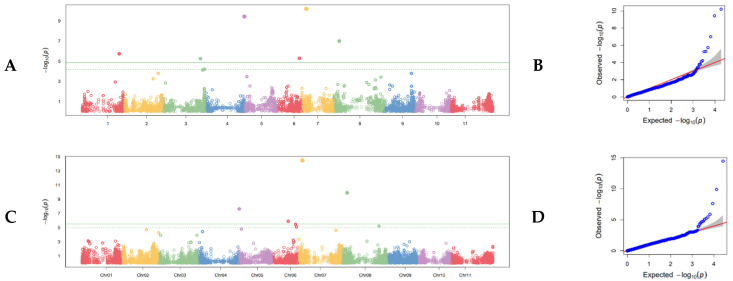
Genome-wide association study of white mold resistance in the SnAP conducted in the greenhouse at Oregon State University using the seedling straw test. (**A**) Manhattan plot of disease severity using the G19833 reference genome with the 11 chromosomes of common bean distributed along the x axis and the (−Log) probability of type I error on the y axis. The cutoff for judging statistical significance of SNPs is the Bonferroni correction of effective marker number at α = 0.05, which is shown as a solid line. The dashed line is an alternative FDR cutoff. (**B**) Q-Q plot corresponding to the Manhattan plot in (**A**). The null distribution is shown as a solid red line. (**C**) Manhattan plot of disease severity using the 5-593 reference genome. Parameters are the same as in A. (**D**). Q-Q plot corresponding to the Manhattan plot in (**C**).

Eleven SNPs distributed across six linkage groups (Pv01, Pv03, Pv05, Pv06, Pv07, and Pv08) showed significant association with white mold resistance (Figure 2A–D; Table 2). The most highly significant SNP was found on Pv07 at position 6,427,041 in the 5-593 genome. Back transformation of the log-value SNP effect for this SNP resulted in an increase of 1.08 on a nine-point scale. The R^2^ according to one-way ANOVA for this SNP was a considerable 0.34. The smallest effect size was associated with the G19833 genome on Pv03 at position 46,795,664, with an effect of −0.03 on a log scale that back transforms to 0.97 on a nine-point scale. This SNP had an R^2^ of 0.01. The combined R^2^ value for all significant SNPs found using the G19833 genome was 0.59, and the combined R^2^ value for all significant SNPs found using the 5-593 genome was 0.49.

A candidate gene search within 100 kb of each SNP position yielded one to three plant-defense-related genes per position (Table 2 and Table S4). Three of the positions were near a pentatricopeptide repeat, four were near a leucine-rich repeat, and two were near an ankyrin gene model. There was also a peroxidase gene model, a PMR5 gene model, a subtilase gene model, and an endoglucanase gene model.

### 3.3. Field Study: Analysis of Variance

Main effects (variety, year, and replication nested within years) were highly significant (*p* < 0.001) for incidence, severity, and DSI (Table 3). In addition, the variety x year interaction was highly significant (*p* < 0.001) for all traits, whereas variety x replication showed no significant differences any of the independent variables. Incidence, severity, and DSI had relatively high R^2^ values, with the model accounting for 89, 79, and 85% of the observed experimental variation, respectively. The coefficients of variation (CVs) for incidence, severity, and DSI were 54.4, 43.1, and 50.2, respectively.

### 3.4. Field Study: Population Distribution

**Figure 3:**
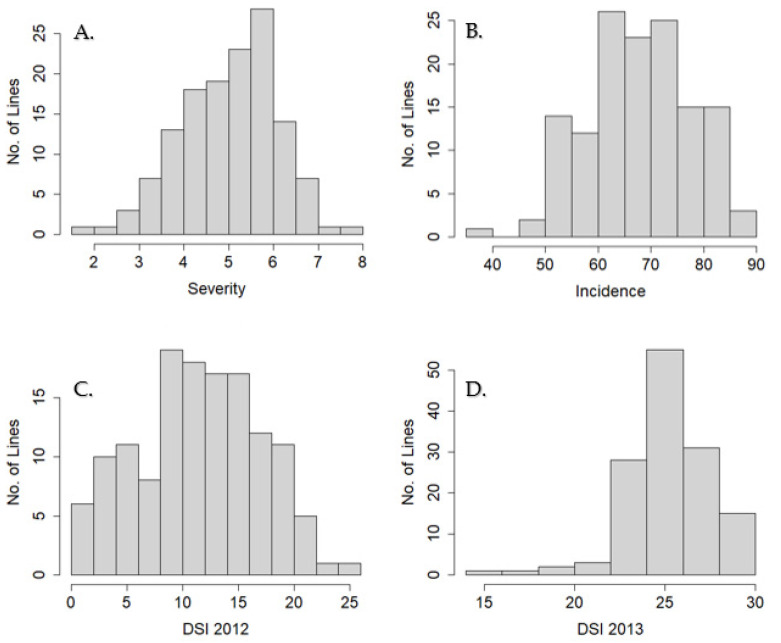
Phenotypic distribution of least square means of traits involved in white mold resistance for the SBDP tested in the field in 2012 and 2013 at the Vegetable Research farm at OSU. (**A**) Severity is reported as the mean of 2012 and 2013 on a scale of 0 to 9. (**B**) Incidence is the mean of 2012 and 2013 on a scale of 0 to 100 percent. (**C**) Disease severity index (DSI) of the geometric mean of severity and incidence in 2012 on a scale of 0 to 30. (**D**) Disease severity index of the geometric mean of severity and incidence in 2013. Lower values indicate more resistance in all four data sets shown.

For resistant traits measured in the field, the LSM of incidence, severity, and DSI of SBDP was calculated for each year. The incidence and severity were averaged across years. Phenotypic distributions were normally distributed, except for a left skew in the DSI 2013 data (Figure 3A–D). For disease severity averaged across years, G122 had the lowest score at 1.7, followed closely by ‘Unidor’ with a score of 1.8 and ‘Venture’ at the opposite extreme, with a score of 8.0 (Table S1). For incidence averaged across years, ‘Unidor’ and G122 had the lowest score, at 38.4, whereas ‘Venture’ had the highest score, at 90 (Table S1). The susceptible check ‘Oregon 91G’ had a disease severity score of 6.3 across both years and an incidence of 73.1 (Table S1). G122 and the resistant experimental lines were all significantly more resistant than the susceptible check, ‘Oregon 91G’.

### 3.5. Field Study: Multiple Correlation among Traits

For disease incidence and severity, Pearson’s correlation coefficients were high for 2012 and moderate for 2013 (0.96 and 0.50, respectively), indicating similarity in ranking for these two disease parameters (Table 4). As would expected, owing to the autocorrelation with incidence and severity, these parameters were highly correlated with DSI in the same year. Lower but significant positive correlations were observed for the disease incidence, severity, and DSI between years, with correlation coefficients of 0.21, 0.44, and 0.42, respectively. Unexpectedly, strong negative correlations were observed between the seedling straw test and disease incidence, severity, and DSI for field tests in 2012, and no significant correlation was observed between the seedling straw test and field traits in 2013 (Table 4).

### 3.6. Field Study: Genome-Wide Association Study (GWAS)

**Figure 4:**
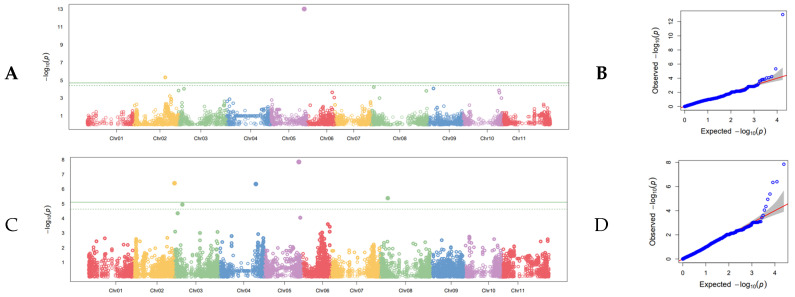
Manhattan plot of combined-years severity GWAS conducted in the field on the SBDP panel. (**A**) Manhattan plot of disease severity using the G19833 reference genome with the 11 chromosomes of common bean distributed along the x axis and the (−Log) probability of type I error on the y axis. The cutoff for judging statistical significance of SNPs is the Bonferroni correction of effective marker number at α = 0.05, which is shown as a solid line. The dashed line is an alternative FDR cutoff. (**B**) Q-Q plot corresponding to the Manhattan plot in (**A**). The null distribution is shown as a solid red line. (**C**) Manhattan plot of disease severity using the 5-593 reference genome. (**D**) Q-Q plot corresponding to the Manhattan plot in (**C**).

**Figure 5:**
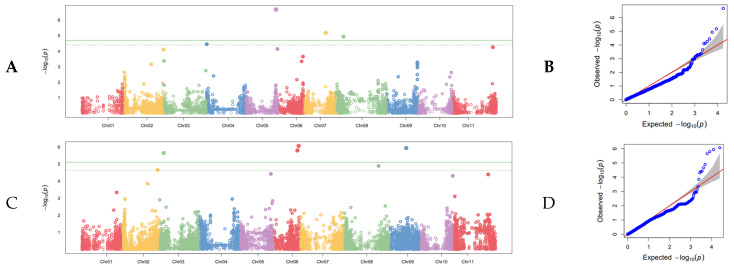
Manhattan plot of combined-years incidence GWAS conducted in the field on the SBDP panel. (**A**) Manhattan plot of incidence using the G19833 reference genome with the 11 chromosomes of common bean distributed along the x axis and the (−Log) probability of type I error on the y axis. The cutoff for judging statistical significance of SNPs is the Bonferroni correction of effective marker number at α = 0.05, which is shown as a solid line. The dashed line is an alternative FDR cutoff. (**B**) Q-Q plot corresponding to the Manhattan plot in (**A**). The null distribution is shown as a solid red line. (**C**) Manhattan plot of disease severity using the 5-593 reference genome. (**D**) Q-Q plot corresponding to the Manhattan plot in (**C**).

**Figure 6:**
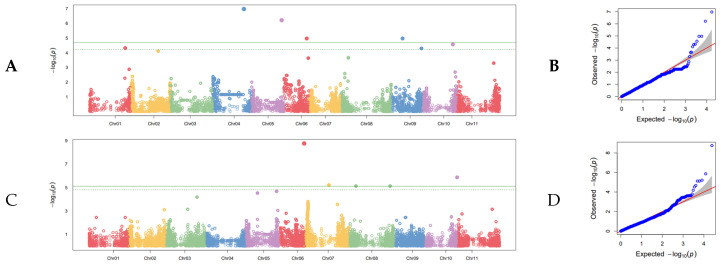
Manhattan plot of DSI 2012 GWAS conducted in the field on the SBDP panel. (**A**) Manhattan plot of incidence using the G19833 reference genome with the 11 chromosomes of common bean distributed along the x axis and the (–Log) probability of type I error on the y axis. The cutoff for judging statistical significance of SNPs is the Bonferroni correction of effective marker number at α = 0.05, which is shown as a solid line. The dashed line is an alternative FDR cutoff. (**B**) Q-Q plot corresponding to the Manhattan plot in (**A**). The null distribution is shown as a solid red line. (**C**) Manhattan plot of disease severity using the 5-593 reference genome. (**D**) Q-Q plot corresponding to the Manhattan plot in (**C**).

**Figure 7:**
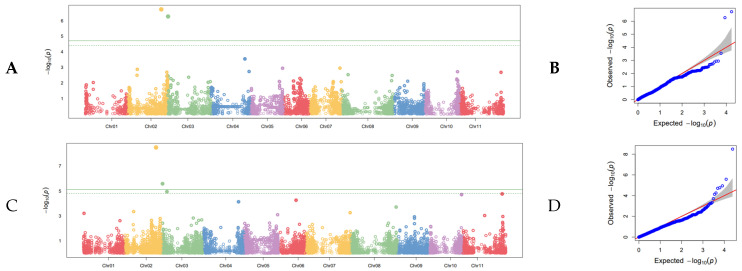
Manhattan plot of DSI 2013 GWAS conducted in the field on the SBDP panel. (**A**) Manhattan plot of incidence using the G19833 reference genome with the 11 chromosomes of common bean distributed along the x axis and the (−Log) probability of type I error on the y axis. The cutoff for judging statistical significance of SNPs is the Bonferroni correction of effective marker number at α = 0.05, which is shown as a solid line. The dashed line is an alternative FDR cutoff. (**B**) Q-Q plot corresponding to the Manhattan plot in (**A**). The null distribution is shown as a solid red line. (**C**) Manhattan plot of disease severity using the 5-593 reference genome. (**D**) Q-Q plot corresponding to the Manhattan plot in (**C**).

An association study conducted on the SBDP for incidence, severity, and DSI identified total of 26 SNP positions, of which 3 were duplicate SNP positions identified in different data sets, for a total of 23 non-duplicated SNP positions (Figure 4, Figure 5, Figure 6 and Figure 7; Table 5, Table 6, Table 7 and Table 8).

Of the 26 SNPs detected in the field GWAS studies, six were found using the combined-years severity data set. Two of these six resulted from applying the G19833 genome to the data set, and four SNPs resulted from applying the 5-593 genome (Figure 4; Table 5). The most significant SNP from the combined years for severity GWAS based on *p*-value was the SNP on Pv05 at position 38,076,792, the G19833 genome, which was also detected when utilizing the combined-years incidence data set and the DSI 2012 data set. However, this SNP did not have the highest R^2^ value as determined in a one-way ANOVA, although the R^2^ value was comparatively high, at 0.14. The highest R^2^ value belonged to the SNP found with the 5-593 genome on Pv08 at position 10,162,589, with an R^2^ value of 0.20. The same SNP on Pv08 was also detected with the DSI 2012 data set. The SNP found with the 5-593 genome on Pv05 at position 43,365,299 also had a relatively high R^2^ of 0.13. The remaining three SNPs had relatively low R^2^ values of 0.02, and one SNP had statistically meaningless R^2^ of <0.01 when analyzed by one-way ANOVA. The combined R^2^ for G19833 was 0.16, and the combined R^2^ for 5-593 was 0.35 in the combined-years severity GWAS.

Of the SNPs for incidence detected in the field GWAS, seven were found with the combined-years incidence data set. Three of these resulted from applying the G19833 genome to the data set, and four resulted from applying the 5-593 genome (Figure 5; Table 6). As with the combined-years severity data set, the most significant SNP detected in the combined-years incidence data set was the SNP on Pv05 at position 38,076,792 of the G19833 genome. In this case, the R^2^ was slightly lower than the combined-years severity data, at 0.09. The highest R^2^ value belonged to the SNP on Pv08 at position 9,597,483, with an R^2^ value of 0.21. The SNP on Pv03 at position 6,274,693, 5-593 also had a relatively high R^2^ of 0.10. Three additional SNPs had R^2^ values ranging from 0.04 to 0.07, and a fourth SNP was a statistically meaningless (<0.01) according to the one-way ANOVA. The combined R^2^ for G19833 was 0.37, and the combined R^2^ for 5-593 was 0.20.

Of the SNPs detected in the field GWAS, nine were found with the DSI 2012 data set. Of these, four were found using the G19833 genome, and five were found with the 5-593 genome (Figure 6; Table 7). The most significant SNP based according to *p*-value was on Pv06 at position 35,297,840, 5-593, and a second SNP on Pv04 at position 38,778,892, G19833, was also highly significant. Despite their low *p*-values, these two SNPs had R^2^ values of only 0.05 and 0.01, respectively. The largest R^2^ value at 0.23 belonged to an SNP on Pv08 at position 10,162,589, 5-593, that is also found in the results of the combined-years severity data set. The SNP on Pv05 at position 38,076,792, G19833, also had a high R^2^ value of 0.10. This SNP on Pv05 is the same SNP that was identified as significant by the combined-years severity and combined-years incidence data sets. The remaining SNPs had R^2^ values ranging from 0.1 to 0.5, with a single SNP at the statistically insignificant value of <0.01. The combined R^2^ value for SNPs detected with the G19833 genome was 0.17, and the combined R^2^ for those detected with 5-593 was 0.33.

Of the SNPs detected in the field GWAS, four were produced by the DSI 2013 data set. Of these, two were associated with the G19833 genome, and two were from the 5-593 genome (Figure 7; Table 8). The most significant SNP was on Pv02 at position 42,951,653, 5-593, and its concomitant R^2^ was also relatively high, at 0.12. The highest R^2^ of 0.20 was associated with an SNP on Pv03 at position 430,964 identified within the G19833 genome. The SNP on Pv03 at position 357,403, 5-593, had a similar R^2^ of 0.19, and it is likely that these SNPs are identical QTL found in different reference genomes. The last SNP on Pv02 at position 41,768,892, G19833, had an R^2^ of 0.12, and is also likely a parallel QTL found in both reference genomes at roughly the same genomic position. Further corroborating the notion that these are the same QTL in different reference genomes is the identical gene annotation found at each SNP position in each reference genome. The SNP on Pv02 occurs within a gene model that is annotated as a T-complex protein 1 subunit (CCT1) in both reference genomes, and the SNP on Pv03 similarly lands within a gene model annotated as an ARM repeat superfamily protein in both reference genomes (Table 8). The combined R^2^ for G19833 is 0.32, and the combined R^2^ for 5-593 is 0.31.

The candidate gene search for all four GWAS of the field data sets revealed one or more plant-defense-related gene models within 100 kb of every significantly associated SNP (Table 5, Table 6, Table 7, Table 8 and Table S4). Twelve non-duplicated SNP positions (52% of the total) were near a pentatricopeptide repeat (PPR). Similarly, nine non-duplicated SNP positions *39% of the total) were near a leucine-rich repeat (LRR), of which two were LRR within a receptor-like kinase (RLK) or serine–threonine kinase (STPK) catalytic domain, with the remainder described as LRR motif genes. Two STPK gene models did not contain LRR motifs. Four SNP positions were near either a DOF zinc finger or a C2H2 zinc finger. Two SNP positions were near an ethylene-responsive transcription factor. One SNP position was near two copies of a chitinase, which may be related to degradation of the chitin-based cell walls of the fungus. Other defense-related proteins that were found near associated SNPs were xyloglucan, thaumatin AT-hook motif, subtilase, thionin, and remorin. Unlike the straw test data that identified two SNPs near ankyrin gene models, no SNPs from the field data were near an ankyrin gene model.

## 4. Discussion

The management of white mold disease through traditional breeding has always represented a significant challenge. Environmental conditions and their interaction with the genotype play an important role in disease development and progression, regardless of physiological resistance. It is generally understood that the straw test is an indicator of physiological resistance, whereas field tests measure physiological resistance in combination with disease avoidance [5,27,28]. The relative importance of these two factors may vary from field trial to field trial depending on growing conditions and production methods. A comparison of the field and greenhouse data reveals significant differences. Differing SNPs are associated with disease severity in these two data sets, and the phenotypic data sets either do not correlate (in 2013) or show negative correlation (in 2012). Other studies of quantitative resistance to white mold have reported low or no correlation between field evaluations and the straw test [5]. According to these differences, field conditions can involve a complex interplay of avoidance factors and physiological resistance. This interaction between genotype and environment may mask the QTL, with small a individual effect associated with physiological resistance.

We examined models with zero, one, two and generally found one PC axis to produce the best fit. A one- or two-PC-axis model makes biological sense and corresponds to the two centers of domestication. A separation of Middle American and Andean-derived accessions occurs along the first PC axis. A second PC axis represents a dispersion of Middle American accessions, varying from Oregon Blue Lake types at one extreme to European Extra Fine types at the other end of the axis, in addition to separating Andean snap beans that possess either T- or C-phaseolin seed storage protein. These may represent independent derivations of snap beans from dry beans, all within the respective centers of domestication [18]. In this study, we found that two PC axes were over fitted and unnecessary.

In the DSI 2012 data set, three SNPs identified by GWAS had unusual allelic ratios. These SNPs were on Pv09 at 14,451,719 bp, Pv07 at 33,701,341 bp, and on Pv10 at 46,178,860 bp. The proportion of heterozygotes detected at these positions was 15% to 24% of the total. This was unexpected in a self-pollinating crop and may represent selective pressure for the heterozygous state or possibly an error in genotyping. None of these three SNPs has a large effect or considerable R^2^ value.

The physical map coordinates for some gene models shifted when the G19833 genome was updated to version 2.1, as reflected by the positions of the six copies of the gene models related to polygalacturonase-inhibiting protein 1 related gene models that exist in the common bean genome (Table 9). This gene is important for white mold resistance [29], although no copies were located near to associated SNPs detected in the present study. In this comparison of gene positions in three different references genomes, not only do the positions shift in all three, but the corrections from V1.0 of G19833 to V2.1 of G19833 can involve shifts between chromosomes (Pv09 to Pv11). Excluding this change of chromosome, the average shift in position for these six gene copies between version 1.0 and version 2.1 of G19833 is 650 kb. The differences in position between the G19833v2.1 genome and 5-593v1.0 range from 1 to 2 Mb. As such, the intervals in which the same gene model may reside may be closer to 500 kb–1 Mb rather than the 100 kb interval that has been used in common bean GWAS research.

The 5-593 and G19833 reference genomes represent different centers of domestication and therefore may have unique gene variants. The SnAP and SBDP panels contain lines from both centers, as well as many lines that are admixtures. Restricting the reference genome for GWAS to an Andean reference genome, such as G19833, may result in missing variants present in the Middle American reference genome, 5-593, and vice versa. In this study, we identified several unique SNPs present only in one of the two reference genomes, although matching identical QTL in both genomes represent a difficulty, owing to the differences in position and sequence. For example, although the SNPs found on Pv02 and Pv03 in both reference genomes for the DSI 2013 data set are likely the same QTL in both genomes (Table 8), the alleles differ in terms of frequencies (A/C versus C/A for the Pv02 QTL) and are different alleles altogether (T/C vs. G/A for the Pv03 QTL).

A recent GWAS study of white mold resistance in a dry bean (*Phaseolus vulgaris*) MAGIC population did not yield any overlaps with our study, although one QTL identified in the current study was adjacent to one reported in [10]. This QTL is located on Pv04 at position 47,083,551 and is 979 kb from the SNP position found in the present study at 46,104,790. The absence of adjacent or overlapping QTL is not entirely surprising. The population used in the dry bean study had a narrow genetic base, consisting of the progeny from an intercross of eight elite pinto bean cultivars and advanced lines of Middle American origin, whereas we used a broad collection of commercial and heritage materials of both Andean and Middle American derivation. Moreover, the use of a relatively generous threshold of 0.1% to identify significant SNPs in the dry bean study may have resulted in the identification of SNPs that were filtered out in the present study by the more conservative Bonferroni cutoff.

A GWAS study of a diversity panel of Spanish common bean accessions produced 15 quantitative trait intervals (QTIs) located on six chromosomes [30]. None of the QTIs in that study overlapped with our QTL, but several were adjacent, including QTI2_34, QTI3_0.9, QTI8_55, and QTI9_16 (Table 10).

In addition to these GWAS studies and an unpublished M.S. thesis [31], genetic mapping of white mold resistance in common bean has been conducted with recombinant inbred line (RIL) populations with relatively large QTL intervals. This work with RIL was summarized by Vasconcellos et al. [9] using the G19833 genome v1.0. The shift between versions of the common bean genome is problematic in terms of making comparisons with the RIL work, but even with sizable shifts in position, it is reasonable to infer overlaps between the QTL identified in the RIL studies and those reported in the present study, owing to the considerably larger intervals of these QTL, with an average length of 5.2 Mb. Half of the straw test SNPs found in the current study overlap with the QTL found in previous RIL studies, and three significantly associated SNPs from the field data overlap (Table 10).

A mix of SNPs ranging from low to relatively high SNP effects and R^2^ values were obtained in this study. This is to be expected for a quantitative trait with numerous contributing loci. This can be extrapolated further through genomic prediction utilizing all SNPs for model construction and selection, even if any individual SNP has an extremely small effect. This is a modeling approach that was not used in the present study, although some SNPs with very small effects were captured through GWAS. Future studies should examine the utility of genomic prediction compared to GWAS.

A high preponderance of PPR genes in close proximity to associated SNPs for white mold resistance has not been noted in previous studies. PPRs are not R genes but are recognized as disease resistance gene analogs [32]. PPRs interact with chloroplasts and mitochondria through modification of organellar transcripts or their processing and translation [33]. Chloroplasts are involved in immune signaling, and both chloroplasts and mitochondria are involved in the production of reactive oxygen species (ROS) and in programmed cell death [32]. The loss of a mitochondrially targeted PPR in Arabidopsis considerably increased susceptibility to necrotrophic fungi from the loss of ROS homeostasis [34]. Research into potato *Ralstonia* infection revealed a differential upregulation of PPRs upon infection [35]. Constitutive expression of a mitochondrially targeted PPR in a mutated rice plant enhanced disease resistance to both fungal and bacterial pathogens [36]. Our research also revealed a high preponderance of LRR type genes, which have long been recognized as disease resistance gene analogs [32,37,38]. LRRs are key molecules in both effector-triggered plant immunity and in pathogen/microbe-associated molecular pattern (PAMP)-triggered immunity [32]. A quarter of the LRRs were STPKs, which are part of a class of transmembrane RLKs. These STPKs are some of the first molecules triggered by PAMP in an immune response cascade [32,39,40]. Not all STPKs possess LRR motifs [39], and two of those identified here did not but may still be involved in pathogen recognition. Collectively, nearly 80% of all SNPs identified in this study were near either a PPR, LRR, or RLK gene, suggesting that these types of defense genes may be of critical importance to building quantitative inheritance of white mold resistance in common bean, specifically snap bean.

The frequency of PPR genes identified in this study is not related to the frequency of this gene family in the genome. A keyword search of the G19833 genome, v2.1, reveals 514 copies of PPR, and a keyword search of the 5-593 genome, v1.1, yields 502 copies. At a total length of 537,218,636 bp, the G19833v2.1 genome would be expected to contain one PPR copy per 1.04 Mb. Likewise, the 5-593v1.0 genome is 572,228,222 bp in length, with one copy expected every 1.14 Mb. Our window for a candidate gene search was far smaller, at 200 kb, which should only yield 6 to 7 copies of the PPR gene if the average distribution were applied to our search windows; however, we found 15 copies near our associated SNPs.

Other defense-related genes were identified in this study. Two ankyrin genes were identified near associated SNPs. Ankyrin is known to be involved in PAMP immune recognition [41]. A study in soybean revealed that *Fusarium* infection upregulated ankyrin gene expression [42]. A gain-of-function mutation in the SNC2 ankyrin gene resulted in constitutive defense responses in *Arabidopsis* [41]. Tandem chitinase genes were also found, and a modification of the chitinase gene or an enhancement of its transcription produced a more robust plant response in degrading chitin cell walls of the white mold fungus [43,44]. Ethylene-responsive transcription factors are also known to be important to plant defense. Ethylene signaling is critical for induced systemic resistance to necrotrophic fungal pathogens, and *Brassica napus* infected with white mold showed induced ethylene pathway activity based on an analysis of transcriptome activity 48 h post infection [7,45]. In addition, zinc finger motif proteins, endoglucanase, subtilase, PMR5, peroxidase, thionin, xyloglucan, remorin, thaumatin, AT-hook, and lipid transfer proteins have all been implicated in plant defense [32,46,47,48,49,50,51,52,53,54,55,56,57,58,59,60,61].

There may be additional candidate genes not identified in this study. Some SNPs may be associated with avoidance traits, and we did not attempt to identify such candidates because avoidance constitutes is a very broad set of traits that are pleiotropically influenced by many genes. The SNP at 46,254,411 on Pv01 is near *fin*, which controls determinacy in common bean. Determinate growth habit has been associated with white mold avoidance, and we screened both determinate and indeterminate accessions in the greenhouse straw test in which this SNP was observed. However, we did not expect the greenhouse straw test to correlate with variation in avoidance traits. Finally, some SNPs might be associated with regulatory genes, and we did not search for these for similar reasons.

## 5. Conclusions

In this study, we identified 34 SNPs associated with white mold resistance from a broad snap bean panel. We used both greenhouse straw tests and field assessments of white mold resistance. Our GWAS models were run with reference genomes from both an Andean and Middle American background, and unique SNPs were identified in each of these, demonstrating the utility of using different reference genomes to tease out associated SNPs that may not be present in some genetic backgrounds. The resulting SNPs were within 100 kb on either side of defense-related genes. The genes for pentatricopeptide repeats and leucine-rich repeats were particularly enriched and may represent an important research avenue with respect to quantitative resistance to white mold.

## Figures and Tables

**Table 1:** Analysis of variance for white mold disease severity in the Snap Bean Association Panel using the seedling straw test in a trial grown in the OSU greenhouse, Corvallis, Oregon, in 2016.

Source	df	Sum of Squares	Mean Square	F Value	Prob. (>F)
**Variety**	377	1103.87	2.928	3.1029	<2.2 × 10^−16^
**Rep**	2	97.20	48.599	51.5008	<2.2 × 10^−16^
**Residual**	754	711.51	0.944		

**Table 2:** SNPs significantly associated with disease severity identified in a GWAS using the seedling straw test of the Snap Bean Association Panel. Reference genome, alleles, chromosome, physical position, *p*-value, negative log *p*-value, minor allele frequency, SNP effect, and R^2^ of SNPs are shown. Multiple copies of the same gene are not shown here but are presented in more detail in Table S4.

Ref. Genome and Version	Alleles	Chr	Position (bp)	*p* Value	Candidate Genes	MAF	Effect	R^2^
G19833, v2.1	C/G	1	46,254,411	1.82 × 10^−6^	PPR	0.05	−0.09	<0.01
G19833, v2.1	A/T	3	46,795,664	5.52 × 10^−6^	PPR	0.23	−0.03	0.01
G19833, v2.1	A/T	5	298,431	3.63 × 10^−10^	Endoglucanase	0.41	0.05	0.21
G19833, v2.1	C/A	6	28,241,345	5.13 × 10^−6^	LRR RLK	0.10	0.05	0.01
G19833, v2.1	T/C	7	5,661,801	6.31 × 10^−11^	LRR RLK	0.34	−0.06	0.34
G19833, v2.1	A/T	8	6,960,113	9.88 × 10^−8^	Subtilase	0.31	0.04	0.02
5-593, v1.0	G/A	5	1,385,913	2.39 × 10^−8^	Ankyrin, LRR	0.48	0.04	0.12
5-593, v1.0	G/A	6	22,888,282	1.3 × 10^−6^	PMR5	0.17	−0.05	0.02
5-593, v1.0	T/G	6	32,949,273	3.51 × 10^−6^	LRR RLK	0.10	0.06	0.01
5-593, v1.0	T/A	7	6,427,041	3.42 × 10^−15^	PPR, peroxidase	0.34	0.07	0.34
5-593, v1.0	G/A	8	7,480,271	1.25 × 10^−10^	Ankyrin	0.28	−0.05	0.01

Chr, chromosome; LRR, leucine-rich repeat, may be combined with RLK, receptor-like kinase; PPR, pentatricopeptide repeat; MAF, minor allele frequency; Effect, log phenotypic effect.

**Table 3:** Analysis of variance for disease incidence, severity, and disease severity index (DSI) of white mold conducted on the SBDP population grown in field trials at the OSU Vegetable Research Farm, Corvallis, Oregon, in 2012 and 2013.

Source	df	Incidence	Severity	DSI
		Mean Sq	Mean Sq	Mean Sq
**Variety**	137	635 ***	7.4 ***	74 ***
**Year**	1	793,242 ***	1588.6 ***	40,543 ***
**Rep (Year)**	4	11681 ***	32 ***	738 ***
Year *Variety	137	531 ***	2.8 ***	34 ***
**Residuals**	540	232 ***	1.5 ***	19 ***

*** = significant at *p* < 0.001

**Table 4:** Pearson’s correlation coefficient analysis of white mold parameters (Inc. = incidence, Sev. = severity, DSI = disease severity index, and SST = seedling straw test) performed on SBDP in the field in 2012 and 2013 and on SnAP in the greenhouse.

	Sev2012	DSI2012	Inc2013	Sev2013	DSI2013	SST
Inc2012	0.96 ***	0.99 ***	0.21 *	0.40 ***	0.41 ***	−0.29 ***
Sev2012		0.99 ***	0.22 **	0.44 ***	0.44 ***	−0.32 ***
DSI2012			0.23 **	0.43 ***	0.43 ***	−0.31 ***
Inc2013				0.50 ***	0.63 ***	0.04 ^ns^
Sev2013					0.99 ***	−0.04 ^ns^
DSI2013						−0.04 ^ns^

* *p* ≤ 0.05; ** *p* ≤ 0.01; *** *p* ≤ 0.001; ns, not significant.

**Table 5:** SNPs significantly associated with combined-years severity. Reference genome, alleles, chromosome, physical position, *p*-value, Negative log *p*-value, minor allele frequency, SNP effect, and R^2^ of SNPs are shown. Multiple copies of the same gene are not shown here but are presented in more detail in Table S4.

Ref. Genome & Version	Alleles	Chr.	Position (bp)	*p*-Value	Candidate Genes	MAF	Effect	R^2^
G19833; v2.1	A/G	2	34,938,882	4.72 × 10^−6^	remorin, PPR, LRR RLK	0.08	−0.39	0.02
G19833; v2.1	C/G	5	38,076,792	9.71 × 10^−14^	zinc finger	0.10	0.84	0.14
5-593; v1.0	T/G	2	51,285,689	3.98 × 10^−07^	PPR	0.17	0.32	0.00
5-593; v1.0	T/C	4	46,104,790	4.55 × 10^−7^	zinc finger	0.15	0.31	0.02
5-593; v1.0	T/C	5	43,365,299	1.41 × 10^−8^	STPK-LRR	0.11	−0.57	0.13
5-593; v1.0	A/T	8	10,162,589	4.18 × 10^−6^	LRR	0.34	−0.27	0.20

Chr, chromosome; LRR, leucine-rich repeat, may be combined with RLK, receptor-like kinase; PPR, pentatricopeptide repeat; STPK, serine–threonine protein kinase; MAF = minor allele frequency; Effect, log phenotypic effect.

**Table 6:** SNPs significantly associated with combined years incidence. Reference genome, alleles, chromosome, physical position, *p*-value, negative log *p*-value, minor allele frequency, SNP effect, and R^2^ of SNPs are shown. Multiple copies of the same gene are not shown here but are presented in more detail in Table S4.

Ref. Genome and Version	Alleles	Chr.	Position (bp)	*p*-Value	Candidate Genes	MAF	Effect	R^2^
G19833; v2.1	C/G	5	38,076,792	2.09 × 10^−7^	zinc finger	0.10	4.74	0.09
G19833; v2.1	G/A	7	27,633,446	6.57 × 10^−6^	PPR	0.07	4.52	0.07
G19833; v2.1	T/A	8	9,597,483	1.14 × 10^−5^	LRR	0.34	−2.62	0.21
5-593; v1.0	A/T	3	6,274,693	2.28 × 10^−6^	PPR, chitinase	0.07	4.69	0.10
5-593; v1.0	C/A	6	33,593,046	1.60 × 10^−6^	STPK, zinc finger	0.15	3.65	0.00
5-593; v1.0	G/A	6	35,277,738	8.75 × 10^−7^	Thionin	0.18	3.48	0.06
5-593; v1.0	G/T	9	23,304,647	1.17 × 10^−6^	PPR	0.06	4.30	0.04

Chr, chromosome; LRR, leucine-rich repeat; PPR, pentatricopeptide repeat; STPK, serine–threonine protein kinase; MAF, minor allele frequency; Effect, log phenotypic effect.

**Table 7:** SNPs significantly associated with DSI 2012. Reference genome, alleles, chromosome, physical position, *p*-value, negative log *p*-value, minor allele frequency, SNP effect, and R^2^ of SNPs are shown. Multiple copies of the same gene are not shown here but are presented in more detail in Table S4.

Ref. Genome and Version	Alleles	Chr.	Position (bp)	*p*-Value	Candidate Genes	MAF	Effect	R^2^
G19833; v2.1	C/T	4	38,778,892	1.07 × 10^−7^	xyloglucan	0.10	2.60	0.01
G19833; v2.1	C/G	5	38,076,792	6.13 × 10^−7^	zinc finger	0.10	2.42	0.10
G19833; v2.1	G/C	6	28,636,391	1.08 × 10^−5^	zinc finger, STPK, PPR	0.16	−1.74	0.02
G19833; v2.1	T/C	9	14,451,719	1.07 × 10^−5^	ER TF, PPR	0.08	2.84	0.04
5-593; v1.0	C/T	6	35,297,840	1.77 × 10^−9^	PPR	0.18	−2.25	0.05
5-593; v1.0	A/C	7	33,701,341	6.19 × 10^−6^	subtilase, LTP	0.33	1.17	0.05
5-593; v1.0	A/T	8	10,162,589	7.54 × 10^−6^	LRR	0.34	−1.30	0.23
5-593; v1.0	G/A	8	57,506,154	7.55 × 10^−6^	AT-Hook, thaumatin	0.15	−1.48	0.01
5-593; v1.0	C/T	10	46,178,860	1.36 × 10^−6^	ER TF, LRR	0.21	−1.93	0.00

Chr, chromosome; LRR, leucine-rich repeat; STPK, serine–threonine protein kinase; PPR, pentatricopeptide repeat; ER TF, ethylene-responsive transcription factor; LTP, lipid transfer protein; MAF, minor allele frequency; Effect, log phenotypic effect.

**Table 8:** SNPs significantly associated with DSI 2013. Reference genome, alleles, chromosome, physical position, *p*-value, negative log *p*-value, minor allele frequency, SNP effect, and R^2^ of SNPs are shown. Multiple copies of the same gene are not shown here but are presented in more detail in Table S4.

Ref. Genome & Version	Alleles	Chr.	Position (bp)	*p*-Value	Candidate Genes	MAF	Effect	R^2^
G19833; v2.1	A/C	2	41,768,892	1.89 × 10^−7^	PPR	0.10	1.40	0.12
G19833; v2.1	T/C	3	430,964	5.38 × 10^−7^	PPR, NBS-LRR	0.14	1.21	0.20
5-593; v1.0	C/A	2	42,951,653	3.25 × 10^−9^	PPR	0.10	1.45	0.12
5-593; v1.0	G/A	3	357,403	2.64 × 10^−6^	PPR, NBS-LRR	0.13	−0.97	0.19

Chr, chromosome; PPR, pentatricopeptide repeat; NBS-LRR, nuclear binding site–leucine-rich repeat; MAF, minor allele frequency; Effect, log phenotypic effect.

**Table 9:** Physical map comparison of gene models related to polygalacturonase-inhibiting protein 1 in three reference genomes. Versions 1.0 and 2.1 of the G19833 genome and version 1.1 of the 5-593 are shown.

Organism	Gene Model	Chrom.	Start (bp)	Stop (bp)
G19833 v1.0	Phvul.001G140000	1	38,799,607	38,800,605
G19833 v1.0	Phvul.002G201600	2	36,090,992	36,092,095
G19833 v1.0	Phvul.002G201700	2	36,100,068	36,101,081
G19833 v1.0	Phvul.002G201800	2	36,117,844	36,119,182
G19833 v1.0	Phvul.002G201900	2	36,135,965	36,137,164
G19833 v1.0	Phvul.009G180400	9	26,512,436	26,521,909
G19833 v2.1	Phvul.001G140000	1	38,150,769	38,151,768
G19833 v2.1	Phvul.002G201600	2	36,740,738	36,741,842
G19833 v2.1	Phvul.002G201700	2	36,749,814	36,750,828
G19833 v2.1	Phvul.002G201800	2	36,767,590	36,768,929
G19833 v2.1	Phvul.002G201900	2	36,785,686	36,786,886
G19833 v2.1	Phvul.011G028800	11	2,612,120	2,615,929
5-593 v1.1	Pv5-593.01G138700	1	41,291,125	41,292,361
5-593 v1.1	Pv5-593.02G196400	2	37,840,455	37,841,463
5-593 v1.1	Pv5-593.02G196500	2	37,849,457	37,850,471
5-593 v1.1	Pv5-593.02G196600	2	37,866,193	37,867,522
5-593 v1.1	Pv5-593.02G196700	2	37,884,415	37,885,717
5-593 v1.1	Pv5-593.11G029800	11	2,998,219	2,999,559

**Table 10:** Overlapping and adjacent QTL between the current study and previous studies These studies included GWAS of a diversity panel [30], QTL analysis of biparental populations [9], and GWAS of a MAGIC population [10]..

Data Set	Ref. Genome	Chr.	Position (bp)	*p* Value	QTL from Other Studies	RI Pop.
Seedling straw test	G19833, v2.1	1	46,254,411	1.82 × 10^−6^	QTI1_45, WM1.1	XC
Mean severity	G19833, v2.1	2	34,938,882	4.72 × 10^−6^	QTI2_34	
DSI 2013	G19833, 5-593	3	430,964	5.38 × 10^−7^	QTI3_0.9	
Seedling straw test	G19833, v2.1	3	46,795,664	5.52 × 10^−6^	WM3.1	XC & AP
Mean severity	5-593, v1.1	4	46,104,790	4.55 × 10^−7^	S04_47083551	
Seedling straw test	5-593, v1.1	6	22,888,282	1.30 × 10^−6^	WM6.1b	R31
DSI 2012	G19833, v2.1	6	28,636,391	1.08 × 10^−5^	WM6.2	XC
Seedling straw test	5-593, v1.1	7	6,427,041	3.42 × 10^−15^	WM7.1	XC & M25
Seedling straw test	G19833, v2.1	8	6,960,113	9.88 × 10^−8^	WM8.3	PS02-029C
Seedling straw test	5-593, v1.1	8	7,480,271	1.25 × 10^−10^	WM8.3	PS02-029C
Mean incidence	G19833, v2.1	8	9,597,483	1.14 × 10^−5^	WM8.3	PS02-029C
DSI 2012	5-593, V1.1	8	10,162,589	7.54 × 10^−6^	WM8.3	PS02-029C
Mean severity	5-593, v1.1	8	10,162,589	4.18 × 10^−6^	WM8.3	PS02-029C
DSI 2012	5-593, v1.1	8	57,506,154	7.55 × 10^−6^	QTI8_55	
DSI 2012	G19833, v2.1	9	14,451,719	1.07 × 10^−5^	QTI9_16	

DSI, disease severity index; Chr., chromosome; QTI#_##, quantative trait intervals from Table 3 of Campa et al. [30]; WM#.#, QTL from Table 2 of Vasconcellos et al. [9]; S04_47083551, significant SNP in Table 3 of Escobar et al. [10]; RI pop, recombinant inbred populations used in biparental QTL analysis in [9]; XC, ‘Xana’ × ‘Cornell 49-242′; AP, ‘AN-37′ × ‘P02630′; PS02-029C, ‘Matterhorn*4′ × ‘NY6020-4′; R31, ‘Raven’ × ‘I9365-31′; M25, ‘Montrose’ × ‘I9365-25′.

## Data Availability

**Supporting data can be found at** ScholarsArchive@OSU. Bean CAP Snap Bean Diversity Panel passport data are available at https://ir.library.oregonstate.edu/concern/datasets/2n49t8455?locale=en (accessed on 1 October 2022).

## Supplementary Materials

The following supporting information can be downloaded at: https://www.mdpi.com/article/10.3390/genes13122297/s1, Table S1: SBDP (Snap Bean Diversity Panel) constituents and mean data for years, combined years, and indexed years; Table S2: SnAP (Snap bean Association Panel) constituents and straw test mean data; Table S3: Shapiro–Wilk test results for mean straw test, mean field data for years, combined field mean years, and indexed field geometric mean years for two snap bean diversity panels; Table S4. Detailed view of candidate genes identified within 100 Kb of an SNP position from GWAS of two snap bean diversity panels.
